# Filaggrin loss-of-function mutations are associated with enhanced expression of IL-1 cytokines in the stratum corneum of patients with atopic dermatitis and in a murine model of filaggrin deficiency

**DOI:** 10.1016/j.jaci.2011.12.989

**Published:** 2012-04

**Authors:** Sanja Kezic, Gráinne M. O’Regan, René Lutter, Ivone Jakasa, Ellen S. Koster, Sean Saunders, Peter Caspers, Patrick M.J.H. Kemperman, Gerwin J. Puppels, Aileen Sandilands, Huijia Chen, Linda E. Campbell, Karin Kroboth, Rosemarie Watson, Padraic G. Fallon, W. H. Irwin McLean, Alan D. Irvine

**Affiliations:** a Coronel Institute of Occupational Health, Academic Medical Center, Amsterdam, The Netherlands; b National Children's Research Centre, Our Lady’s Children’s Hospital, Dublin, Ireland; c Department of Paediatric Dermatology, Our Lady’s Children’s Hospital, Dublin, Ireland; d Departments of Experimental Immunology and Respiratory Medicine, Academic Medical Centre and University of Amsterdam, Amsterdam, The Netherlands; e Laboratory for Analytical Chemistry, Department of Chemistry and Biochemistry, Faculty of Food Technology and Biotechnology, University of Zagreb, Zagreb, Croatia; f Center for Optical Diagnostics and Therapy, Department of Dermatology, Erasmus MC, Rotterdam, The Netherlands; g River Diagnostics BV, Rotterdam, The Netherlands; h Department of Dermatology, Erasmus MC, Rotterdam, The Netherlands; i Epithelial Genetics Group, Medical Sciences Institute, University of Dundee, Dundee, United Kingdom; j Department of Medicine, Trinity College, Dublin, Ireland

**Keywords:** Atopic dermatitis, confocal Raman spectroscopy, eczema, filaggrin, natural moisturizing factor, pH, transepidermal water loss, IL-1, IL-18, skin barrier, *FLG* gene mutations, *Flg^ft^/Flg^ft^* (*Flg^delAPfal^*) mice, AD, Atopic dermatitis, AD_*FLG*_, AD with *FLG* mutations, AD_*NON-FLG*_, AD without *FLG* mutations, *FLG*, Filaggrin gene, *FLG*^−/−^, Patient homozygous for null alleles (ie, 2 null alleles), *FLG*^+/−^, Heterozygote null allele/wild-type (ie, 1 null allele), *FLG*^+/+^, Homozygote wild-type (ie, 0 null alleles), IL-1RA, IL-1 receptor antagonist, NESS, Nottingham Eczema Severity Score, NMF, Natural moisturizing factor, SC, Stratum corneum, TEWL, Transepidermal water loss

## Abstract

**Background:**

Filaggrin *(FLG)* mutations result in reduced stratum corneum (SC) natural moisturizing factor (NMF) components and consequent increased SC pH. Because higher pH activates SC protease activity, we hypothesized an enhanced release of proinflammatory IL-1 cytokines from corneocytes in patients with atopic dermatitis (AD) with *FLG* mutations (AD_*FLG*_) compared with that seen in patients with AD without these mutations (AD_*NON-FLG*_).

**Objectives:**

We sought to investigate SC IL-1 cytokine profiles in the uninvolved skin of controls and patients with AD_*FLG*_ versus patients with AD_*NON-FLG*_. We also sought to examine the same profiles in a murine model of filaggrin deficiency (*Flg^ft^/Flg^ft^* [*Flg^delAPfal^*] mice).

**Methods:**

One hundred thirty-seven patients were studied. NMF levels were ascertained using confocal Raman spectroscopy; transepidermal water loss and skin surface pH were measured. IL-1α, IL-1β, IL-18, IL-1 receptor antagonist (IL-1RA), and IL-8 levels were determined in SC tape strips from 93 patients. All subjects were screened for 9 *FLG* mutations. *Flg^ft^/Flg^ft^ (Flg^delAPfal^)* mice, separated from *maFlg^ft^/maFlg^ft^* (flaky tail) mice, were used for the preparation and culture of primary murine keratinocytes and as a source of murine skin. RT-PCR was performed using primers specific for murine IL-1α, IL-1β, and IL-1RA.

**Results:**

SC IL-1 levels were increased in patients with AD_*FLG*_; these levels were inversely correlated with NMF levels. NMF values were also inversely correlated with skin surface pH. Skin and keratinocytes from *Flg^ft^/Flg^ft^* mice had upregulated expression of IL-1β and IL-1RA mRNA.

**Conclusions:**

AD_*FLG*_ is associated with an increased SC IL-1 cytokine profile; this profile is also seen in a murine homologue of filaggrin deficiency. These findings might have importance in understanding the influence of *FLG* mutations on the inflammasome in the pathogenesis of AD and help individualize therapeutic approaches.

The recent identification of mutations in the gene encoding the key epidermal protein filaggrin *(FLG)* as a remarkably strong and widely replicated risk factor for atopic dermatitis (AD) has led to a new focus on skin barrier deficiency in patients with AD. However, the functional consequences of *FLG* mutations and the downstream mechanisms that underlie immunologic changes in AD skin remain largely unknown.^1,2^

Before the discovery of *FLG* mutations, Elias and Feingold^3^ hypothesized a barrier abnormality as a driving force for development of an inflammatory response in patients with AD. This so-called outside-inside hypothesis is in contrast to a more traditional view known as the inside-outside hypothesis, which holds that skin barrier defects in patients with AD are a secondary consequence of the inflammatory response to irritants and allergens.^4^ Elias et al^5-7^ subsequently hypothesized that reduced levels of filaggrin and in particular its acidic derivative urocanic acid lead to increased pH of the stratum corneum (SC), altering the activity of the multiple serine proteases and 2 ceramide-generating enzymes that regulate homeostasis of the SC.^7^ Another important downstream consequence of increased pH and serine protease activity is generation of the active primary cytokines IL-1α and IL-1β from their inactive proproteins, representing the first step in the cytokine cascade that has been proposed as a primary contributor to inflammation in patients with AD. Sustained antigen ingress through a defective barrier leading to a T_H_2-dominant infiltrate is proposed as a secondary cause of inflammation in patients with AD.

Mediators from the IL-1 gene family control innate immune responses through a number of mechanisms, including promoting the recruitment of leukocytes and regulating synthesis of the extracellular lipid bilayers as the principal barrier of the skin.^5,8,9^ IL-1 mediators also bridge the innate and adaptive immune systems and thus constitute an important function in immune defense.^10,11^ There are 11 members of the IL-1 family of which IL-1α, IL-1β, IL-1 receptor antagonist (IL-1RA), and IL-18 have been most thoroughly studied. IL-1α and IL-1β initiate responses by binding to the IL-1 receptor, which is antagonized by IL-1RA. These elements of the IL-1 system are represented in the epidermis. Keratinocytes constitutively produce high amounts of IL-1α, and the epidermis contains important quantities of biologically active preformed IL-1α.^12,13^ In addition, in inflammatory conditions human keratinocytes also produce IL-1β^14^; however, blood monocytes, tissue macrophages, and dendritic cells are considered the primary sources of IL-1β.^10^ IL-1α is produced in the cytoplasm as a precursor protein (pro–IL-1α) and processed into a mature protein by the intracellular calcium–dependent cysteine protease calpain, which has an optimum activity at neutral pH.^12,15^ The precursor of IL-1β is biologically inactive and must be cleaved into its biologically active form, a process that is largely mediated by caspase-1, although some serine proteases and several other enzymes have been reported to cleave both IL-1α and IL-1β.^10,12,16,17^ Caspase-1 is a cysteine protease that also cleaves precursors of IL-18, a cytokine that plays an important role in the pathogenesis of AD.^18,19^ The pH optimum for caspases ranges between 6.5 and 6.8.^20^ Thus multiple proteases crucial for SC homeostasis and cleavage of IL-1 cytokines have optimal activity at pH values higher than the physiologic outer SC/skin surface layer pH.^5-7^ Release of IL-1 cytokines leads to cutaneous inflammation through the induction of secondary cytokines, such as IL-8, and upregulation of endothelial adhesion molecules.^10,12^ Production of IL-1β has also been linked to the sensitization and initiation phase of contact allergy.^21,22^

Although little is known about the very early events initiating atopic skin inflammation, it is likely that primary proinflammatory cytokines play an important role. Although several research groups have investigated IL-1 cytokines in the lesional and noninvolved skin of patients with AD,^22-24^ thus far, no study has focused on cytokine levels in patients with AD in relation to *FLG* genotype. Using Raman spectroscopy, we have recently shown that *FLG* genotype is a major determinant of natural moisturizing factor (NMF) in the SC.^25,26^ In the present study we sought to determine the levels of IL-1 cytokines in the SC of uninvolved skin and to relate these levels to *FLG* genotype, pH, and levels of filaggrin degradation products, which are the constituents of NMF. Furthermore, in a complementary murine study we examined the effects of filaggrin status on IL-1 expression in the skin and isolated keratinocytes.

## Methods

### Clinical study: Subjects

One hundred thirty-seven unrelated Irish children with a history of moderate-to-severe AD were recruited from dedicated secondary and tertiary referral AD clinics; the collection used in this study overlaps but is not identical to that used for previous studies.^26^ Diagnosis and phenotyping of AD was made by experienced pediatric dermatologists. All subjects met the United Kingdom diagnostic criteria.^27^ Exclusion criteria from the study were patients who had received systemic therapy, such as oral corticosteroids or immunosuppressants, in the preceding 3 months and patients whose ancestry was not exclusively Irish (4/4 grandparents). Detailed phenotypic data were collected and are presented in Table I. The Nottingham Eczema Severity Score (NESS)^28^ was selected as a measure of chronic disease severity. The study was conducted in accordance with Helsinki Declarations and was approved by the Research Ethics Committee of Our Lady’s Children’s Hospital, Dublin, Ireland. Full written consent was obtained from all patients or their parents.

### Genotyping

All patients were screened for the 9 most common *FLG* mutations found in the Irish population (R501X, Y2092X, 2282del4, R2447X, S3247X, R3419X, 3702X, S1040X, and G1139X) and as previously described.^26^ These 9 mutations account for greater than 95% of all *FLG* mutations in the Irish population (Irvine and McLean, unpublished data). On the basis of screening for these 9 prevalent mutations, 59% were carriers of 1 or more *FLG* mutations (42% *FLG*^+/+^ [homozygote wild-type; ie, 0 null alleles], 40% *FLG*^+/−^ [heterozygote null allele/wild-type; ie, 1 null allele], and 18% *FLG*^−/−^ [patient homozygous for null alleles; ie, 2 null alleles]).

### Determination of cytokines in the SC

The levels of cytokines in the tape strips were measured in the SC of the volar forearm by using a previously described method.^29^ Briefly, round adhesive tape discs (3.8 cm^2^, D-Squame; CuDerm, Dallas, Tex) were attached to the skin of the forearm. Each tape was pressed onto the volar aspect of the forearm for 10 seconds with standardized force by using a disc pressure applicator (CuDerm).^30^ The tape strip was gently removed with tweezers and stored in a closed vial at −80°C until analysis. The first strip was discarded because it might have contained dirt and remnants of cosmetic products; the second, third, and fourth tape strips were applied on the same skin spot. The collected 3 tape strips were cut into 2 equal pieces. For the analysis, halves of 3 strips were pooled for the analysis. To determine the amount of soluble protein and cytokines, 2 mL of PBS (Merck, Darmstadt, Germany) with 0.005% Tween-20 (Sigma-Aldrich, Zwijndrecht, The Netherlands) was added to each vial, and the vials were left on ice for 30 minutes. Extraction was performed with an ultrasound sonifier equipped with a probe (Salm & Kipp, Breukelen, The Netherlands) for 15 minutes in ice water. The extract was centrifuged (for 1 minute at 15,000*g*), and supernatant aliquots of 225 μL were refrozen at −80°C until required for further analysis.

Concentrations of IL-1α, IL-1β, IL-1RA, and IL-8 in the SC strips were measured with a Luminex-based multiplex system (Bio-Plex Human Cytokine 27-plex panel and single plexes; Bio-Rad Laboratories, Hercules, Calif) on a Bioplex 100, according to the manufacturer’s instructions. Samples were diluted 4-fold. The amount of cytokines was normalized for the protein content, which was determined by using the Micro BCA protein assay kit (Pierce, Rockford, Ill), with the BSA supplied as standard. For statistical analysis, cytokine concentrations of less than the limit of detection were given a value of half the limit of detection. The limit of detection was 0.2 pg/mL for IL-1α, 0.01 pg/mL for IL-1β, 1.4 pg/mL for IL-1RA, 0.3 pg/mL for IL-8, and 0.045 pg/mL for IL-18.

### Biophysical analysis of the SC

Skin biophysical measurements were performed under standardized conditions (room temperature of 22°C-25°C and humidity levels of 30% to 35%). Before measurements, patients were acclimatized for a minimum of 10 minutes. All measurements were performed by one of 2 investigators (G. M. O'R. and P. M. J. H. K.). Topical therapies, including emollients, were withheld from the measurement sites for 48 hours preceding the study. Transepidermal water loss (TEWL) and pH were measured on nonlesional skin of the extensor forearm (Tewameter 300 and Skin-pH-Meter, PH905; Courage and Khazaka Electronic GmbH, Cologne, Germany).

NMF was measured in the SC of the thenar eminence by using confocal Raman microspectroscopy (model 3510 Skin Composition Analyzer; River Diagnostics, Rotterdam, The Netherlands). The principles of this method and the procedure have been described elsewhere.^25,26^

In a further 17 adult control subjects and 25 white patients with AD, we determined the levels of IL-1α by using a specific ELISA kit (Biosource International, Camarillo, Calif). Healthy volunteers had no visible skin abnormalities or history of past or present AD or other skin diseases. All of them were wild-type for the 4 most common *FLG* mutations in the Dutch population (R501X, 2282del4, R2447X, and S3247X). Patients with AD were given diagnoses according to the Hanifin and Rajka criteria^31,32^ and were divided into 2 subgroups according to the presence of *FLG* mutations. We excluded patients who had received systemic therapy, such as corticosteroids and immunosuppressants, or phototherapy in the past 3 months. The test sites, both midvolar arms, had been free of dermatitis for at least 3 months before the experiment. Written informed consent was obtained from all subjects before participation. The study was approved by the Ethics Committee of the Academic Medical Center, Amsterdam, and was conducted according to the principles of the Declaration of Helsinki. Nine of the patients with AD were wild-type for *FLG* mutations (AD_*NON-FLG*_), and 12 were heterozygous and 4 were homozygous or compound heterozygous carriers of 4 investigated mutations (AD_*FLG*_).

### Murine study

Adult (12- to 14-week-old) C57BL/6J or congenic *Flg^ft^/Flg^ft^ (Flg^delAPfal^)* mice, separated from *maFlg^ft^/maFlg^ft^* (flaky tail) mice, were used for the preparation and culture of primary murine keratinocytes.^33^ These mice are homozygous for murine *Flg* loss-of-function mutations and express no filaggrin protein.^33^ Mice were housed in a pathogen-free facility in individually ventilated and filtered cages under negative pressure (Tecniplast, Northants, United Kingdom). All animal experiments were performed in compliance with Irish Department of Health and Children's regulations and approved by the Trinity College Dublin BioResources Ethical Review Committee.

### Murine keratinocyte culture

Isolation of murine primary keratinocytes was performed as previously described.^34,35^ Briefly, the epidermis was separated from the dermis of tail skin after overnight incubation in 0.25% trypsin (Lonza, Basel, Switzerland). Keratinocytes were isolated from the SC with trituration, centrifuged at 150*g*, and filtered through a 100-μm sieve (BD Biosciences, San Jose, Calif), followed by further centrifugation. The cells were resuspended in calcium-free KGM-2 media (BulletKit, Lonza) and adjusted to 0.05 mmol/L Ca^2+^, with 50 U/mL penicillin and 50 μg/mL streptomycin (Gibco, Carlsbad, Calif). Cells were plated on fibronectin/collagen-coated dishes and incubated at 36°C at 7% CO_2_. The keratinocytes were cultured for 4 days, at which point the attached keratinocytes were analyzed for expression of cytokine mRNA or induced to differentiate. Cells were cultured in KGM-2 BulletKit media adjusted to 0.5 mmol/L Ca^2+^ and harvested for analysis at 72 hours to induce keratinocytes to undergo terminal differentiation.

### RNA isolation and real-time PCR

RNA was isolated from primary keratinocytes, prepared as above, or dorsal skin from mice by using the RNeasy kit (Qiagen, Crawley, United Kingdom) and reverse transcribed with the Quantitect reverse transcription kit incorporating a genomic DNA elimination step (Qiagen). Real-time quantitative PCR was performed on an ABI Prism 7900HT sequence detection system (Applied Biosystems, Paisley, United Kingdom) using predesigned TaqMan gene expression assays specific for murine IL-1α (Mm00439620_m1), IL-1β (Mm01336189_m1), and IL-1RA (IL-1rn;Mm00446186_m1). Specific gene expression was normalized to murine glyceraldehyde-3-phosphate dehydrogenase. Fold expression was calculated by using the comparative cycle threshold method of analysis and is presented as relative quantification.^36^ Data expressed as relative quantification were calculated and compared with glyceraldehyde-3-phosphate dehydrogenase as a housekeeping gene.

### Statistical analysis

Patients were characterized *a priori* into 3 genotypes (*FLG*^+/+^*, FLG*^+/−^, and *FLG*^−/−^), where *FLG*^+/+^ represents patients with 0 *FLG* mutations, *FLG*^+/−^ represents patients with 1 *FLG* mutation (heterozygotes), and *FLG*^−/−^ represents patients with 2 *FLG* mutations (homozygotes or compound heterozygotes). For testing of distribution, we used the Shapiro-Wilk test. Data on cytokine levels were log-transformed and presented as medians with interquartile ranges (25th-75th box length). For comparison of differences between the genotype subgroups of patients with AD, ANOVA followed by a *post hoc* Tukey analysis was applied. In case of deviation from normal distribution, we used the Kruskal-Wallis and Dunn multiple comparison tests. *P* values of less than .05 were considered statistically significant. For the correlation analysis, we used the Spearman rank correlation test in the case of deviation from normal distribution. The Student *t* test was used to test for statistical differences between wild-type and *Flg^ft^/Flg^ft^* mice in cytokine mRNA expression in isolated skin or keratinocytes. The Prism 5 (GraphPad Software, Inc, San Diego, Calif) and SPSS (17.0; SPSS, Inc, Chicago, Ill) software programs were used for statistical calculations.

## Results

### Patients' characteristics

Clinical characteristics and summary data of the study cohort, including *FLG* genotype, are outlined in Table I. The 3 *FLG* genotype subgroups had similar disease severity, as assessed by using the NESS. TEWL values, which are one measure of skin barrier function, were not significantly different among the 3 AD subgroups (*P* > .05, Kruskal-Wallis test). Although there was an apparent increase in IgE levels in patients with AD with respect to the number of *FLG* mutations (Table I), there was no significant difference in total IgE levels between the *FLG* subgroups. *FLG* mutations were associated with reduced levels of NMF (Table I). The difference between patients with AD_*FLG*_ and patients with AD_*NON-FLG*_ was highly (*P* < .001) significant, as was the difference between the *FLG*^+/+^ and *FLG*^−/−^ (*P* < .001) and *FLG*^+/+^ and *FLG*^+/−^ (*P* < .05, Kruskal-Wallis test followed by Dunn Multiple comparison test) subgroups. The amounts of extracellular protein harvested by using tape strips did not significantly differ between patient groups with and without *FLG* mutations (data not shown). Because the study took some time to complete, not all data points were collected from all patients. Patient numbers collected for each analysis are shown in Table I.

### Cytokine levels in relation to *FLG* allele status and NMF

**Fig 1:**
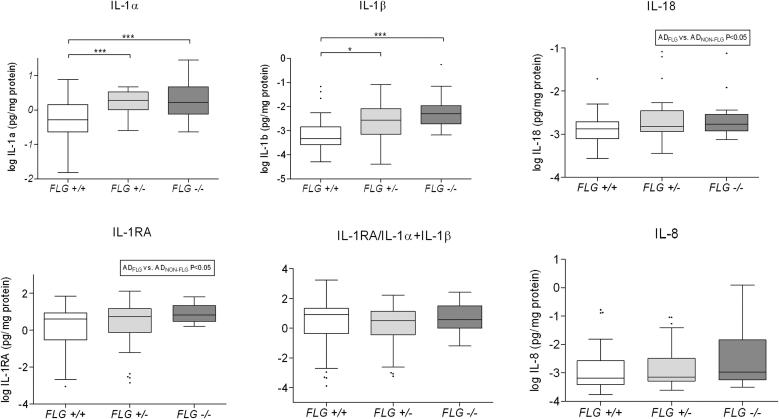
Box-and-whiskers plot cytokine levels in the SC by *FLG* genotype showing the median *(midline)* and interquartile ranges. ∗*P* < .05 and ∗∗∗*P* < .001, as determined by using Kruskal-Wallis and Dunn multiple comparison tests. The differences in IL-18 and IL-1RA levels between the AD_NON-FLG_ and AD_FLG_ groups have been tested by using the 2-sided Mann-Whitney test.

Patients with AD_*FLG*_ had higher amounts of IL-1α and IL-1β compared with patients with AD_*NON-FLG*_ (Fig 1). The differences in IL-1RA and IL-18 levels between the 3 subgroups did not reach significance, as assessed by using Dunn multiple comparison testing. However, a statistically significant difference for both IL-1RA and IL-18 levels was observed between carriers of *FLG* null mutations (AD_*FLG*_ and AD_*NON-FLG*_: *P* < .05 and *P* = .02, respectively, Mann-Whitney 2-sided test). Levels of IL-8 and the IL-1RA/IL-1α plus IL-1β ratio were not significantly different between the *FLG* subgroups nor between the patients with AD_*FLG*_ and AD_*NON-FLG*_.

**Fig 2:**
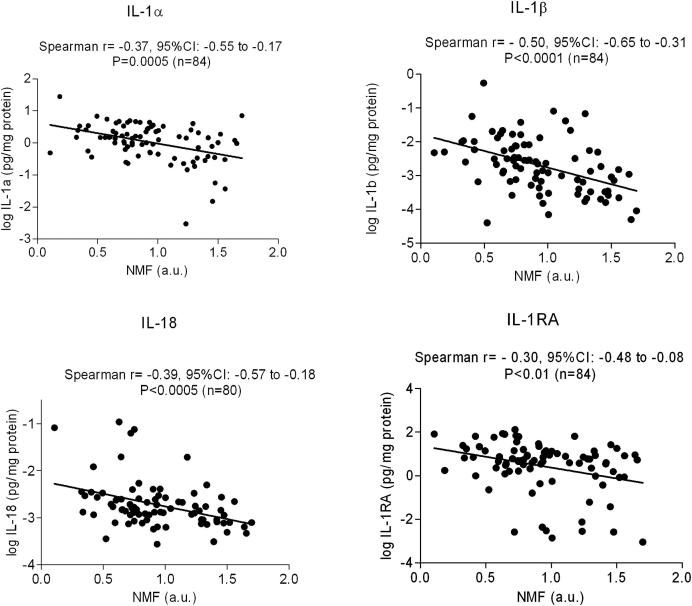
Correlation between levels of cytokines and NMF in the SC of patients with AD. Correlations are unadjusted for *FLG* mutations. *a.u*., Arbitrary units; *r*, Spearman (2-tailed) correlation coefficient.

Correlations between IL-1 cytokines, IL-8 levels, and clinical parameters are further shown in Table II. Levels of IL-18 were associated with those of IL-1β; however, neither IL-18 nor IL-1β levels were correlated with IL-1α levels. Interestingly, IL-1β and IL-18 levels, but not IL-1α levels, were positively correlated with IL-1RA and IL-8 levels (Table II). The IL-1RA/IL-1α plus IL-1β ratio was positively correlated with IL-18 and IL-8 levels; disease severity, as measured based on the NESS; TEWL; and IgE levels. We did not observe an association between pH and IL-1 cytokine levels (Table II). Correlations between NMF and IL-1 cytokine levels are shown in Fig 2 and Table III; the NMF level was inversely associated with levels of all investigated IL-1 cytokines.

**Fig E1:**
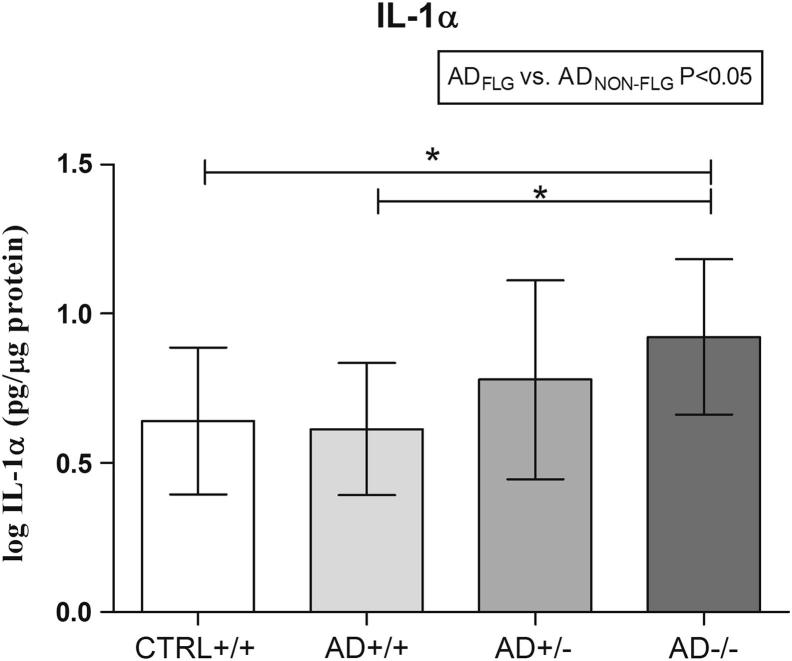
Levels of IL-1α in the SC of healthy control subjects and patients with AD *FLG* genotype. The differences have been tested by using the Student *t* test (∗*P* < .05). Data are presented as mean values ± SDs. *CTRL+/+*, Homozygote wild-type subject without AD.

In the small additional adult study, levels of IL-1α in the SC of patients with AD_*FLG*_ were significantly higher than those in healthy control subjects and patients with AD_*NON-FLG*_ (see Fig E1 in this article's Online Repository at www.jacionline.org).

### SC pH in relation to *FLG* allele status

**Fig 3:**
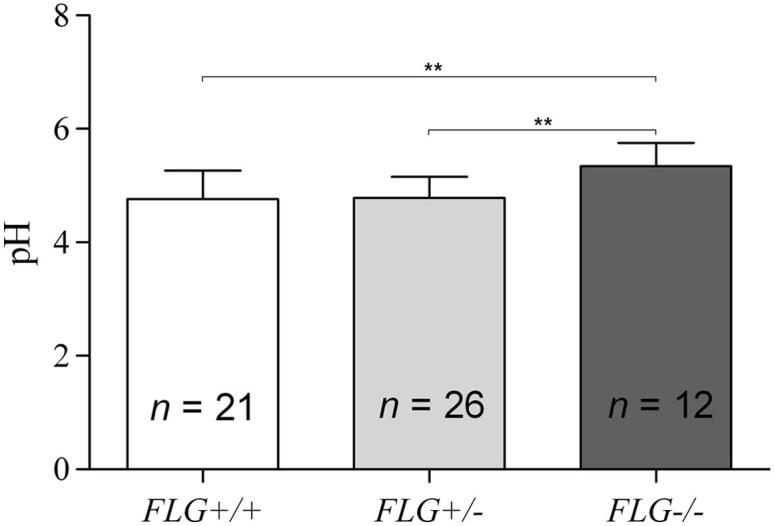
Distribution of pH values according to *FLG* status (mean ± SD). ∗∗*P* < .01, as determined by using the ANOVA *post hoc* Tukey test.

**Fig 4:**
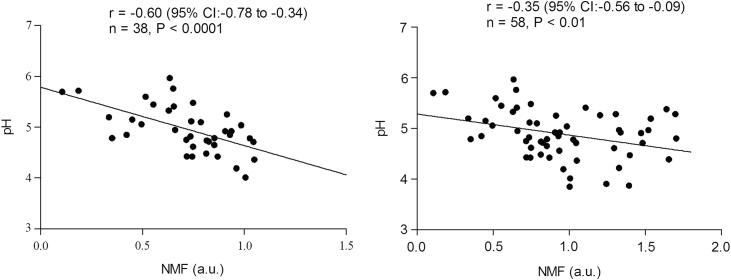
Skin pH values in relation to NMF levels. *Left*, Patients with AD_*FLG*_. *Right*, Patients with AD_*FLG*_ plus patients with AD_*NON-FLG*_. *a.u*., Arbitrary units; *r*, Pearson (2-tailed) correlation coefficient.

Homozygous and compound heterozygous carriers (*FLG*^−/−^) had higher pH values than wild-type (*FLG*^+/+^) or heterozygous (*FLG*^+/−^) carriers (Fig 3). Levels of NMF in the SC of the patients with AD were negatively correlated with skin surface pH, as shown in Fig 4. The correlation was more striking in the subgroup of *FLG* mutation carriers (Fig 4, left graph) compared with all subjects (Fig 4, right graph).

### Animal study in Flg^ft^/Flg^ft^ (Flg^delAPfal^) mice

**Fig 5:**
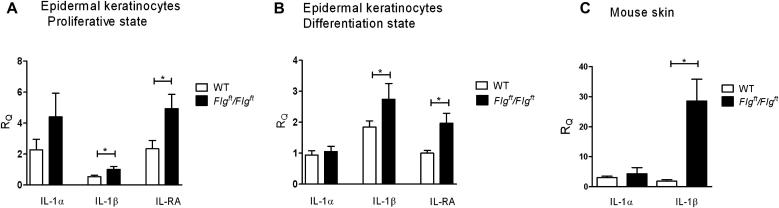
**A** and **B**, Expression of IL-1α and IL-1β in keratinocytes and skin from *Flg^ft^/Flg^ft^* mice. mRNA expression of IL-1β and IL-1RA in primary epidermal keratinocytes in the proliferative (Fig 5, *A*) and terminal differentiation (Fig 5, *B*) states. **C**, IL-1α and IL-1β mRNA expression in dorsal murine skin. Data are representative of 3 independent experiments (means ± SEMs). ∗*P* < .05.

Skin and keratinocytes from *Flg^ft^/Flg^ft^* mice have upregulated expression of IL-1β and IL-1RA mRNA. mRNA expression of IL-1β and IL-1RA is upregulated in primary epidermal keratinocytes in the proliferative (Fig 5, *A*) and terminal differentiation (Fig 5, *B*) states, but there was no significant change in levels of IL-1α. Dorsal skin from *Flg^ft^/Flg^ft^* mice had increased IL-1β mRNA expression but not IL-1α expression relative to levels detected in wild-type animals (Fig 5, *C*).

## Discussion

In this study we sought to investigate whether levels of IL-1 cytokines, including IL-1α, IL-1β, IL-18, and IL-1RA, are increased in the uninvolved skin of patients with AD with *FLG* loss-of-function mutations. Although we have recently shown that AD severity *per se* influences SC NMF status,^37^ this is a minor effect compared with the major effect that *FLG* genotype has on NMF.^26,37^ Because AD severity was similar across all 3 *FLG* genotype subgroups, this AD severity effect is unlikely to influence the results presented herein.

We found increased IL-1α, IL-1β, IL-18, and IL-1RA expression in patients with AD *FLG* mutations compared with that seen in wild-type patients with AD. In line with these results, IL-1 cytokine levels correlated inversely with NMF levels in the SC. In a complementary murine study we also observed altered mRNA IL-1 expression in the skin and cultured keratinocytes of mice with an *Flg* loss-of-function mutation.

Our findings support the hypothesis by Elias et al^5-7^ that “reduced levels of filaggrin and in particular its acidic derivatives such as urocanic acid, lead to increased pH of the SC, which may promote activity of serine proteases involved in cleavage of the pro-forms of IL-1α and IL-1β.”^7,16,17^ Here we show a correlation between increased pH and decreased NMF levels, which is consistent with recent data. An increased (less acidic) pH can activate proteases with neutral-to-alkaline pH optima to process inactive forms of IL-1α, IL-1β, and IL-18 that are stored in the cytosol of corneocytes.^16,38-41^ Consistent with this view, Hosomi et al^42^ showed increased activity of caspase-1 in the SC of patients with Netherton syndrome, an autosomal recessive inherited disease characterized by features of AD and uninhibited breakdown of filaggrin in the SC. Increased pH in the *FLG*^−/−^ genotype subgroup compared with the *FLG*^+/+^ and *FLG*^+/−^ subgroups and the negative correlation between NMF and pH demonstrated in the present study support the view that NMF contributes to the pH of the SC. This is in good agreement with a recent study by Jungersted et al.^43^ However, we found no correlation between SC pH and the SC levels of either IL-1α or IL-1β, but this might be due to a lack of power in our study to determine this correlation. It should be noted that we measured pH on the skin surface and that this measurement might not be representative of the epidermal compartment, where proforms of IL-1 cytokines are activated. Furthermore, activity of proteases and consequently levels of IL-1 might be influenced also by factors other than pH, such as increased calcium concentration caused by reduced water in the SC. We showed previously that carriers of *FLG* mutations have reduced levels of NMF, which likely contribute to reduced SC water content.^25,26,30^ The activity of serine proteases is calcium dependent, and thus increased calcium concentrations caused by decreased cytosolic water content could favor their activation.^44-46^ Proteolytic activity can be influenced further by exogenous proteases, such as those derived from house dust mite, cockroach, and *Staphylococcus aureus*. Allergens derived from house dust mites and *S aureus* exhibit cysteine and serine protease activity.^47-51^ Consistent with these findings, Inoue et al^52^ reported increased levels of a caspase-1–mediated IL-18 in the SC of patients with AD; IL-18 production was associated with *S aureus* colonization. Recently, we have shown that the presence of filaggrin breakdown products results in reduced growth rates of *S aureus* and decreased expression of several proteins known to be involved in colonization and inflammation of skin.^53^ Thus reduced levels of filaggrin degradation products might lead to the increased growth of *S aureus* and consequently to enhanced activity of proteases in the skin.

In the murine study, using *Flg^ft^/Flg^ft^* mice, upregulated expression of IL-1β and IL-1RA mRNA was seen in skin and isolated keratinocytes. No difference in IL-1α expression was detected. The murine results suggest that loss-of-function mutations in *FLG* are a determinant of increased epidermal IL-1β expression. In the murine study we measured mRNA, which should not be influenced by SC pH-enhancing protease activity, and thus a pH-driven increase in protease activity is unlikely to entirely explain the increased IL-1β levels in these mice.

Filaggrin has a key role in aggregation of the keratin filaments in the corneocytes and is likely to play a role in the integrity of the SC through other indirect effects.^1,2^ Barrier disruption itself stimulates keratinocyte proliferation, as well as cytokine and chemokine production, including IL-1α and IL-1β.^54-56^ However, as shown by us and others, the skin barrier in patients with AD is impaired irrespective of *FLG* mutation status,^26,43,57^ and thus barrier dysfunction *sensu latu* would not appear to explain differences in IL-1 cytokine levels between patients with AD with and without *FLG* mutations. Our data suggest that there is an *FLG*-specific effect on the IL-1 cytokine profile. Our small pilot study, which includes healthy adult control subjects (see Fig E1), also supports an *FLG*-specific effect rather than a general AD effect. In this small study control subjects had similar IL-1α levels to those seen in patients with AD_*NON-FLG*_, whereas among patients with AD, those with *FLG* mutations had increased IL-1α levels.

IL-1α and IL-1β are potent proinflammatory mediators, but their activity is counteracted by IL-1RA, levels of which were also increased in patients with AD_*FLG*_. The IL-1RA/IL-1α plus IL-1β ratio was similar in all 3 *FLG* subgroups. Levels of IL-1β and IL-18, but not IL-1α, were positively correlated with levels of IL-1RA and IL-8. This might indicate that in patients with AD_*FLG*_, IL-1β and IL-18 drives inflammation, although we cannot exclude other underlying proinflammatory stimuli. The nature of this proinflammatory stimulus remains to be determined, but it might be independent of the variation in skin pH. For example, it is known that IL-4 and IL-13, which are overexpressed in unaffected AD skin,^58-60^ can drive IL-1RA expression. These cytokines, when coexpressed, have a further inhibitory effect on *FLG* expression,^61^ and thus a positive feedback loop might be in place.

Taken together, our human and murine data support the concept that there might be a pre-existing enhanced or proinflammatory status in the skin of patients with AD that relates to *FLG* mutations. Additional work is required to clarify the temporal link between *FLG* mutations and the enhanced amounts of these proinflammatory cytokines in human subjects, but the murine work presented herein suggests that it is an early effect. The major findings for IL-1β are paralleled by those for IL-18, which share the same maturation and secretory mechanism. It would be interesting to investigate whether subjects with *FLG* mutations but without a history of AD also have increased amounts of IL-1 cytokines.

**Key messages**

• Among patients with AD, those with AD_*FLG*_ have a distinct SC cytokine profile from those with AD_*NON-FLG*_.
• Similar cytokine profile findings were replicated in a murine homologue of filaggrin deficiency.
• This is the first study to link *FLG* mutations with alterations in IL-1 levels.

## Figures and Tables

**Table I:** Cohort characteristics according to genotype

*FLG* genotype†	All mutations combined	Age (y), median (range)	Male sex, no. (%)	NESS, median (range)	TEWL (g/m^2^/h), median (range)	Log IgE,∗ mean (SD)	NMF (AU), median (range)	pH, mean (SD)
Screened (n)	137	137	137	137	99	131	129	58
+/+	57 (41.6%)	8 (3-17)	34 (59.6%)	12 (4-15)	12.6 (5.2-47.4), n = 39	2.9 (1.0), n = 54	1.33 (0.73-1.65), n = 51	4.75 (0.51), n = 20
+/−	55 (40.1%)	7 (1-17)	32 (58.2%)	12 (4-15)	14.05 (5.3-33.0), n = 43	2.8 (0.8), n = 54	0.83 (0.40-1.29), n = 54	4.79 (0.37), n = 26
−/−	25 (18.2%)	8 (3-17)	15 (60.0%)	12 (6-15)	15.9 (8.5-36.1), n = 17	3.1 (0.7), n = 23	0.58 (0.18-0.76), n = 24	5.34 (0.41), n = 12
*P* value		.84‡	.97§	.36‡	.22‡	.45‖	<.0001‖	<.0006¶

*AU*, Arbitrary unit.

∗ IgE data were positively skewed. The mean of the log-transformed data is presented.

† +/+ denotes no *FLG* mutation, +/− denotes 1 *FLG* mutation, and −/− denotes 2 *FLG* mutations.

‡ Comparison of means among the 3 groups of *FLG* mutations using the Kruskal-Wallis test.

§ Comparison of proportions using the χ^2^ test for comparison of a 2 × 3 × 3 contingency table.

‖ Comparison of means among the 3 groups of *FLG* mutations using ANOVA.

¶ ANOVA.

**Table II:** Correlations between IL-1 cytokine levels, IL-8 levels, and clinical parameters

	IL-1α	IL-1β	IL-18 (n = 87)	IL-8 (n = 91)	NESS (n = 92)	TEWL (n = 78)	IgE (n = 86)	pH (n = 40)
IL-1α		NS	NS	NS	−0.30 (−0.48 to −0.09), *P* < .005	NS	NS	NS
IL-1β	NS		0.41 (0.21 to 0.57), *P* < .005	0.31 (0.10 to 0.49), *P* < .005	NS	0.32 (0.10 to 0.51), *P* < .005	NS	NS
IL-18	NS	0.41 (0.21 to 0.57), *P* < .005		0.38 (0.18 to 0.55), *P* < .0005	NS	0.33 (0.11 to 0.52), *P* < .005	NS	NS
IL-1RA	NS	0.50 (0.33 to 0.65), *P* < .0001	0.50 (0.32 to 0.65), *P* < .0001	0.32 (0.11 to 0.49), *P* < .005	0.31 (0.10 to 0.48), *P* < .005	0.42 (0.21 to 0.60), *P* = .0001	NS	NS
IL-1RA/IL-1α plus IL-1β	−0.64 (−0.75 to −0.49), *P* = .02	0.33 (0.13 to 0.51), *P* = .001	0.40 (0.20 to 0.57), *P* < .0001	0.24 (0.04 to 0.44), *P* = .02	0.39 (0.19 to 0.55), *P* = .0002	0.39 (0.19 to 0.55), *P* < .0001	0.26 (0.04 to 0.45), *P* < .05	NS

Results are presented as Spearman correlation coefficient values (2-tailed) with 95% CIs and significance.

*NS*, Not significant.

**Table III:** Correlations between NMF, cytokine levels, and clinical parameters

	IL-1α (n = 84)	IL-1β (n = 84)	IL-18 (n = 80)	IL-1RA (n = 84)	IL-8 (n = 83)	NESS (n = 129)	TEWL (n = 99)	IgE (n = 126)	pH (n = 58)
NMF	−0.37 (−0.55 to −0.17), *P* = .0005	−0.50 (−0.65 to −0.31), *P* < .0001	−0.39 (−0.57 to −0.18), *P* < .0005	−0.30 (−0.48 to −0.08), *P* < .01	NS	NS	−0.35 (−0.51 to −0.15), *P* = .0004	NS	−0.35 (−0.56 to −0.09), *P* < .01

Results are presented as Spearman correlation coefficient values (2-tailed) with 95% CIs and significance.

*NS*, Not significant.
